# Polyamine Metabolism Is Involved in the Direct Regeneration of Shoots from Arabidopsis Lateral Root Primordia

**DOI:** 10.3390/plants10020305

**Published:** 2021-02-05

**Authors:** Nikolett Kaszler, Péter Benkő, Dóra Bernula, Ágnes Szepesi, Attila Fehér, Katalin Gémes

**Affiliations:** 1 Institute of Plant Biology, Biological Research Centre, Hungarian Academy of Sciences, 62. Temesvári krt, H-6726 Szeged, Hungary; kaszler.nikolett@brc.hu (N.K.); benko.peter@brc.hu (P.B.); bernula.dora@brc.hu (D.B.); 2 Doctoral School of Biology, University of Szeged, 52. Közép fasor, H-6726 Szeged, Hungary; 3 Department of Plant Biology, University of Szeged, 52. Közép fasor, H-6726 Szeged, Hungary; szepesia@bio.u-szeged.hu

**Keywords:** *Arabidopsis thaliana*, hydrogen peroxide, polyamines, polyamine oxidase, reactive oxygen species, direct shoot regeneration, spermidine

## Abstract

Plants can be regenerated from various explants/tissues via de novo shoot meristem formation. Most of these regeneration pathways are indirect and involve callus formation. Besides plant hormones, the role of polyamines (PAs) has been implicated in these processes. Interestingly, the lateral root primordia (LRPs) of Arabidopsis can be directly converted to shoot meristems by exogenous cytokinin application. In this system, no callus formation takes place. We report that the level of PAs, especially that of spermidine (Spd), increased during meristem conversion and the application of exogenous Spd improved its efficiency. The high endogenous Spd level could be due to enhanced synthesis as indicated by the augmented relative expression of PA synthesis genes (*AtADC1,2, AtSAMDC2,4, AtSPDS1,2*) during the process. However, the effect of PAs on shoot meristem formation might also be dependent on their catabolism. The expression of Arabidopsis POLYAMINE OXIDASE 5 (*AtPAO5*) was shown to be specifically high during the process and its ectopic overexpression increased the LRP-to-shoot conversion efficiency. This was correlated with Spd accumulation in the roots and ROS accumulation in the converting LRPs. The potential ways how PAO5 may influence direct shoot organogenesis from Arabidopsis LRPs are discussed.

## 1. Introduction

De novo organogenesis from somatic plant tissues occurs both in nature or in vitro, either directly or indirectly through callus formation [1]. During these processes, explants or calli first form ectopic apical meristems, which subsequently develop into shoots or roots, respectively. Among the plant hormones, auxin and cytokinin are the most important to regulate plant morphogenesis including organ formation. Based on the recognition that high auxin to cytokinin ratios promote root while high cytokinin to auxin ratios shoot formation, the establishment of in vitro plant regeneration systems were elaborated for hundreds of plant species. In the model plant, *Arabidopsis thaliana*, shoot regeneration is usually achieved via indirect organogenesis from root explants [2,3,4]. In recent years, it has been recognized that callus formation is not required for de novo shoot formation from root tissues and the direct conversion of lateral root primordia (LRP) to shoot meristem can take place in response to cytokinin application [5,6,7]. Three successive phases of this process could be distinguished [7]. First, exogenous cytokinin transiently pauses cell division in the LRPs (mitotic pause) and the regulators of shoot development start to be expressed. This is followed by the conversion phase in which the root meristem is converted into a shoot promeristem with a characteristic cell division pattern. Finally, the promeristem matures and develops into a fully functional shoot meristem.

Auxin and cytokinin interact with other plant growth regulators, including polyamines (PAs) during several physiological and developmental processes [8,9,10]. PAs are highly reactive polycations. The three most common PAs in plants are the diamine putrescine (put), the triamine spermidine (Spd), and the tetramine spermine (spm), although other PAs, such as cadaverine and thermospermine (t-spm), are also present in plants [11]. Several studies indicated that PA metabolic and catabolic pathways are linked to plant growth and development [12]. Among others, polyamines have been reported to be involved in a variety of morphogenic processes such as organogenesis and somatic embryogenesis [13,14,15,16].

In plants, Put is synthesized by two pathways: either directly from ornithine via ORNITHINE DECARBOXYLASE (ODC; EC 4.1.1.17) or indirectly from arginine via ARGININE DECARBOXYLASE (ADC; EC 4.1.1.19). Interestingly, in *Arabidospsis thaliana* there is no ODC and only the ADC-catalyzed pathway contributes to Put biosynthesis [17]. S-ADENOSYLMETHIONINE DECARBOXYLASEs (SAMDCs) catalyze the formation of an important intermediate of spermidine and spermine biosynthesis, the decarboxylated S-adenosylmethionine (dSAM). The Arabidopsis double mutant *samdc1 samdc4*/*bud2* not expressing two SAMDC isoenzymes is embryo lethal, while the single *samdc4*/*bud2* mutant has a bushy dwarf phenotype demonstrating that SAMDCs are essential for plant development and morphogenesis [18]. It was found that the *bud2* mutation results in auxin and cytokinin hypersensitivity suggesting that polyamines may interfere with these plant hormones regulating plant growth [19]. Moreover, t-Spm has recently been identified as a novel plant growth regulator (PGR) which represses xylem differentiation and promotes stem elongation [8,20,21,22]. T-Spm is synthesized by thermospermine synthase ACAULIS5 (ACL5) and *acl5* is allelic to *thickvein* (*tkv*), which displays defective auxin transport [23,24]. Furthermore, t-Spm potently suppresses the expression of genes involved in auxin signaling, transport, and synthesis [25].

Besides PA synthesis, PA catabolism is also connected to cellular signaling during developmental processes via the generation of H_2_O_2_ [26]. In cotton, PAs and their catabolic product H_2_O_2_ were found to be essential for the conversion of embryogenic calli into somatic embryos [27]. Polyamine catabolism is mediated mainly by two classes of amine oxidases. One of them is DIAMINE OXIDASE (DAO) which oxidizes mainly Put and the other is the apoplastic POLYAMINE OXIDASE (PAO), which preferentially oxidizes Spd and Spm but not Put yielding aminoaldehydes and H_2_O_2_ [28,29]. In cotton somatic embryogenesis, the H_2_O_2_ concentration augmented during the formation of embryogenic calli in parallel with the increased expression of GhPAO1 and GhPAO4 and the overall PAO activity [27].

In addition to their terminal oxidation, PAO enzymes catalyze the backconversion of tetraamine to triamine and/or triamine to diamine [9,30]. In the Arabidopsis genome, there are five PAO genes (*AtPAO1–AtPAO5*) [30]. Two of them (*AtPAO1* and *AtPAO5*) are localized in the cytoplasm and involved in the catabolism/backconversion of Spd, Spm and t-Spm. *AtPAO1* and *AtPAO5* both catalyze the backconversion reaction of Spm and t-Spm to Spd. However, AtPAO5 unlike the other Arabidopsis PAOs including AtPAO1 has a dehydrogenase rather than oxidase activity [9,31]. Therefore, it was hypothesized that AtPAO5 exerts its regulatory role during plant growth and xylem differentiation maintaining polyamine homeostasis rather than increasing hydrogen peroxide production [8,9]. AtPAO5 has a high affinity for t-Spm and it was hypothesized to contribute to the tightly regulated interplay of auxin and cytokinin during xylem differentiation and plant growth via controlling the t-Spm level [8,9]. The remaining three AtPAOs of Arabidopsis (AtPAO2, AtPAO3, and AtPAO4) have a peroxisomal localization and can oxidize/backconvert both Spd and spm, but not t-Spm [30,32,33,34,35].

Although, the involvement of polyamines in indirect organogenesis was investigated in several studies, the impact of their regulatory role on direct organogenesis has not been shown yet. In this study, our aim was to investigate the involvement of PAs and polyamine metabolism in the direct conversion of lateral root primordia into shoot meristems.

## 2. Materials and Methods

### 2.1. Plant Materials and Culture Conditions

*Arabidopsis thaliana* wild type (WT) plants of the ecotype Columbia (Col-0) were used along with *AtPAO2* (*35S:PAO2*) and *AtPAO5* transgenic plants (*35S::PAO5*) previously described [8,9,30,31]. To induce direct organogenesis the method of Rosspopoff et al. [7] was applied with minor modifications. All plants were grown in a climate-controlled cabinet using 8/16 (light/dark) photoperiod and a constant temperature of 21 °C with an irradiance of 50 µmolm^−2^s^−1^ provided by white fluorescent tubes (Sylvania Luxline Plus; Feilo Sylvania Europe Limited, London, UK). Surface-sterilized seeds (70% ethanol for 60 s followed by immersion in 4% commercial sodium hypochlorite solution (having 4.5% active chlorine) for 10 min) were sown and grown for 6 days on solid medium containing full-strength MS (Murashige and Skoog Medium including B5 vitamins, Duchefa Biochemie, Haarlem, The Netherlands), 1% sucrose (Molar Chemicals, Halásztelek, Hungary), 0.6 % agarose (Electran DNA pure grade for electrophoresis: VWR International LLC, Radnor, PA, USA), 0.5 g/L 2-(*N*-morpholino)ethanesulfonic acid (MES) (Duchefa Biochemie). To induce lateral root (LR) initiation 3.3 µM naphthaleneacetic acid (NAA) (Duchefa Biochemie) priming was applied for 43 h. For synchronization of LR initiation, 1.25 µM 2,3,5-triiodobenzoic acid (TIBA) (Fluka, Chemie GmbH, Buchs, Switzerland) was added to the germination medium before the NAA treatment. To induce the conversion of lateral root primordia (LRP) into a functional shoot meristem (SM), seedlings were transferred onto and cultured on a medium (SM medium) containing full-strength MS salts (Duchefa Biochemie), 2% D(+)-glucose (Molar Chemicals), 0.6 % agarose (Electran DNA pure grade for electrophoresis: VWR International LLC, Radnor, PA, USA) and 8.16 µM isopentenyl-adenine (IPA) (Sigma-Aldrich, St. Louis, MO, USA). Stock solution of spermidine and putrescine, respectively, was prepared in Milli-Q^®^ H_2_O, filter sterilized with Millex^®^ GV syringe filter (0.22 µm), (Merck Millipore, Burlington, MA, USA) and added to the SM medium at 100 µM final concentration. For sample collection, roots of seedlings were used after 48, 72 and 96 h cytokinin induction (at the 96th hour of induction the seedlings were ≈12 days old). As absolute control, roots of 6-days-old, nontreated seedlings were used. For gene expression analysis, samples were harvested and snap-frozen in liquid N_2_ and they were stored at −80 °C until usage.

### 2.2. Gene Expression Analysis

For total RNA extraction Quick-RNA Miniprep Kit (Zymo Research, Irvine, CA, USA) was used which includes the removal of contaminating genomic DNA. NanoDrop™ 2000/2000c spectrophotometer (Thermo Fisher Scientific, Waltham, MA, USA) was used to evaluate the quality and quantity of total isolated RNA, considering the ideal absorbance ratio (1.8 ≤ A260/280 ≤ 2.0). In total, 250–300 ng of total RNA was reverse-transcribed for 60 min at 42 °C and for 10 min at 75 °C in a 20 μL reaction volume using RevertAid First Strand cDNA Synthesis Kit (Thermo Fisher Scientific) according to the manufacturer’s instructions. cDNA products were diluted 1:10 in AccuGENE^®^ water (Lonza, Verviers, Belgium). Primers were designed using Primer 3 software [36] and synthesized by Biocenter Ltd. (Szeged, Hungary). Primer sequences are shown in Supplementary Table S1. Primer sequences were analyzed using OligoAnalyzer^TM^ Tool (Integrated DNA Technologies, Inc., Coralville, IA, USA) and National Center for Biotechnology Information (NCBI) programs (Bethesda (MD): National Library of Medicine (US), National Center for Biotechnology, 1982). Relative mRNA levels were determined by real-time quantitative PCR (RT-qPCR). As reference genes, *UBIQUITIN 1* (*At3G52590)* and PP2A3 *(At1G13320)*) were used. These genes were selected using the Arabidopsis Regeneration eFP browser at The Bio-Analytic Resource for Plant Biology (bar.u-toronto.ca; [37]) allowing the in silico analysis of transcriptomic data sets of root-to-shoot regeneration experiments. According to these data, the *At3G52590* and *At1G13320.1* genes have constitutive expression during the process. The RT-qPCR reactions were carried out by the qTOWER 2.0 (Analytic Jena AG, Life Science, Jena, Germany) and CFX384 Touch Real-Time PCR Detection System (Bio-Rad Laboratories Inc., Hercules, CA, USA). Depending on the detection system, the PCR mixture contained (in a total volume of 7 or 14 μL) 1 or 2 μL cDNA, 0.21 or 0.42 μL forward primer, 0.21 or 0.42 μL reverse primer, 3.5 or 7 μL Maxima SYBR Green/ROX qPCR Master Mix (2×) (Thermo Fisher Scientific). Reaction mixture was aliquoted to 96-well plates (non-skirted, white; Thermo Fisher Scientific, Cat no: AB-0600/W) or Hard-Shell^®^ 384-well plates (thin-wall, skirted, clear/white; Bio-Rad, Cat. no: HSP3805). For amplification, a standard two-step thermal cycling profile was used (10 s at 95 °C and 1 min at 60 °C) during 40 cycles, after a 15 min preheating step at 95 °C. Finally, a dissociation stage was added with 95 °C for 15 s, 60 °C for 15 s and 95 °C for 15 s. Data analysis was performed using qPCRsoft (Analytic Jena, AG), Bio-Rad CFX Maestro (Bio-Rad) software and Microsoft Excel 2010. The relative mRNA levels normalized to the average of *AT3G52590* and *AT1G13320.1* mRNAs were calculated using the (2)^−ΔΔCt^ method. The mRNA level of the initial root tissue was used as control (relative mRNA level: 1). All tested amplification efficiencies were in a narrow range and were not used in the data normalization. Data were averaged from three independent biological experiments with three technical replicates for each gene/sample combination.

### 2.3. Light Microscopy

The number of regenerated plantlets on seedling roots were determined using an Olympus SZX12 stereo dissection microscope (Olympus Corporation, Sindzsuku, Tokyo, Japan). For the bright field images, white LED light source (Photonic Optics, Vienna, Austria) was used. Photos were captured using an Olympus Camedia C7070 digital camera and the DScaler software (version 4.1.15).

### 2.4. In Situ Detection of Reactive Oxygen Species

For in situ detection of ROS, 2,7-dichlorodihydrofluorescein diacetate (H_2_DC-FDA, Sigma-Aldrich, St. Louis, MO, USA) was applied [38,39]. Seedling roots were incubated in 10 μM H2DC-FDA solubilized in 2-Nmorpholine-ethansulphonic acid/potassium chloride (MES/KCl) pH 6.5 for 15 min at room temperature in darkness. After staining, seedling roots were washed once with a dye-free buffer and the fluorescence of the oxidized product of H_2_DC-FDA, dichlorofluorescein (DCF), was visualized by a fluorescent microscope. To detect fluorescence intensity, a Zeiss Axiowert 200 M-type fluorescent microscope (Carl Zeiss, Germany) equipped with a high-resolution digital camera (Axiocam HR) was used. The fluorescence intensity was estimated by the Axiovision Rel. 4.8 software using a filter set 10 (excitation occurred at 450−490 nm and emission was detected at 515−565 nm). The same camera settings were used for each digital image. Means of pixel intensities were calculated within each image within a 40 μm diameter circle of the converting organ (after 48 h cytokinin induction), early and late shoot promeristem (72 and 96 h after cytokinin induction) (d = 40 μm). The relative fluorescence intensities of at least 20 converting organs/shoot promeristems in each of three replicates were measured using Image J, and mean relative fluorescence intensities were calculated.

### 2.5. Free Polyamine Determination

Free PA contents were determined as described by [40]. In brief, 100 mg of whole root samples were homogenized in 5% perchloric acid. After centrifugation, 0.5 mL of the supernatant was neutralized with 0.4 mL of 2 M NaOH, then the PAs were derivatized with 10 µL of benzoyl chloride. The benzoylated polyamines were separated by HPLC system (JASCO, HPLC system, Japan) equipped with reverse-phase C18 column (Apex octadecyl, 5 µm; 4.6 mm × 250 mm), eluent was 45% (*v*/*v*) acetonitrile/water, with flow rate at 0.5 mL/min monitored with UV detector at 254 nm. Then, 20 µL injection of standards and samples were analyzed. The applied standards were Put, Spd, and Spm in the form of hydrochlorides (Sigma-Aldrich, Germany). The results are the means of three independent biological samples expressed in nmol g^−1^ fresh weight^−1^.

### 2.6. Statistical Analysis and Data Representation

Statistical analysis was performed using SIGMAPLOT12.0 statistical software. Quantitative data are presented as the mean ± SE and the significance of difference between sets of data was determined by one-way analysis of variance (ANOVA) following Duncan’s multiple range tests; *p*-values of less than 0.05 were considered significant. In some cases, Student’s *t*-test was used as indicated (* *p* ≤ 0.05, ** *p* ≤ 0.01, *** *p* ≤ 0.001).

## 3. Results and Discussion

### 3.1. PA Accumulation Correlates with and Promotes the Formation of Shoot Meristems from Lateral Root Primordia

To investigate the potential involvement of polyamines in the process of direct shoot regeneration, the PA content was determined in cytokinin-treated Arabidopsis roots at 0, 48, 72, and 96 h. These time points mark three main stages of the cytokinin-induced conversion of lateral root primordia (LRP) into a shoot meristem [7]. Following a cytokinin induced mitotic pause of ≈24 h, the LRPs go through a conversion phase (sampled at 48 h) and gradually develop to early (72 h) and late (96 h) shoot promeristems [7].

**Figure 1:**
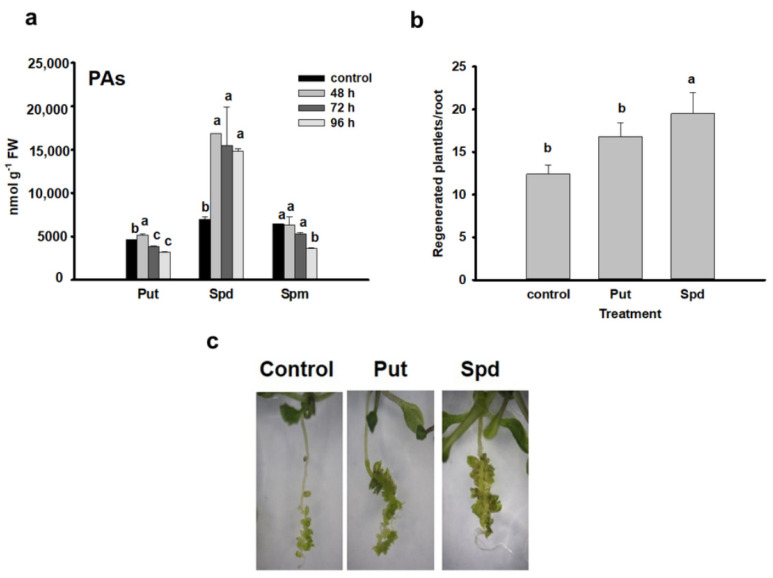
Polyamines are involved in the conversion of lateral root primordia into shoot meristem. (**a**) Changes in the polyamine content after 48, 72 and 96 h of cytokinin induction of Arabidopsis Scheme 48 h), and the early (72 h) and late (96 h) shoot promeristem phases [7]. (**b**) Effect of exogenously applied 100 µM spermidine and putrescine, respectively, on the efficiency of shoot regeneration of wild-type Col seedling roots determined 10 days after cytokinin induction. Images of representative seedlings are shown in (**c**) Data are means ± SE of three biological replicates with 20 technical replicates each. Different letters indicate significant differences of Duncan’s multiple comparisons (*p* < 0.05).

The level of the three main polyamines changed differently during the cytokinin-induced meristem conversion and development process (Figure 1a). The level of putrescine exhibited a slight but significant transient increase at 48 h, followed by decrease at later time points. The amount of spermine slightly but gradually decreased during the process. In contrast, the spermidine concentration exhibited a considerable (≈3-fold) increase in all three time points as compared to the control.

Exogenous application of Put and especially Spd enhanced the shoot regeneration efficiency of Arabidopsis seedling roots which further verified the positive effect of these polyamines on direct organogenesis (Figure 1b,c).

The levels of endogenous polyamines have been often found to correlate with the in vitro regeneration efficiency of explants and exogenous polyamines were successfully used in many cases to enhance indirect somatic embryogenesis or shoot organogenesis in various species in a concentration and genotype-dependent manner [14,15,41,42]. In most of these experiments, Put and Spd were used.

Since ethylene synthesis is metabolically linked to the accumulation of PAs as both utilize the same intermediate, *S*-adenosylmethionine (SAM), it was hypothesized that PA-dependent morphogenic responses might be associated with changes in ethylene levels [16]. Ethylene is a potent inhibitor of in vitro plant regeneration and the enhancement of shoot regeneration by ethylene inhibitors was attributed to accumulation of PAs in *Brassica alboglabra* explants [43]. Conversion of Put to Spd or Spm involves the addition of aminopropyl moieties from decarboxylated S-adenosylmethionine (dSAM). When Spd and Spm synthesis was blocked inhibiting the SAM DECARBOXYLASE (SAMDC) enzyme, shoot regeneration was prevented in correlation with elevated ethylene production (Cheng, 2002 in Pua, 2007). However, shoot regeneration could be restored to normal level using exogenous PAs without diminishing the ethylene level indicating the direct role of PAs in shoot regeneration. In agreement, inhibition of *SAMDC* expression by RNA interference in transgenic Arabidopsis strongly promoted shoot regeneration increasing the Spd+spm/Put ratio but not affecting the ethylene level [44].

In accordance with previous observations [44,45], we found that a high endogenous Spd/Put ratio correlates with shoot regeneration and both PAs promoted shoot organogenesis if exogenously applied. To have a better insight into the regulation of endogenous PA levels during the direct shoot organogenesis process, the expression of several genes coding for enzymes implicated in PA metabolism has been investigated in cytokinin-treated roots during the process of LRP-to-SM conversion.

### 3.2. Put and Spd Synthesis Is Augmented during the Meristem Conversion Process

**Figure 2:**
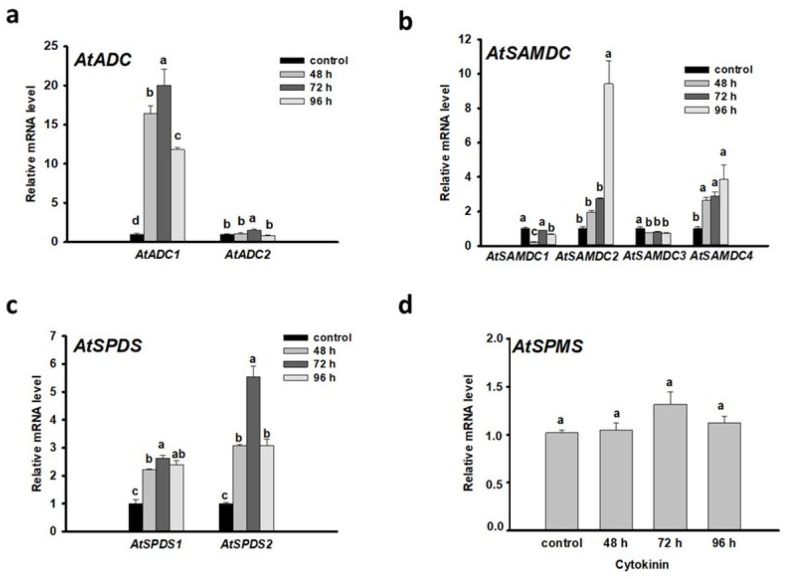
Expression of genes coding for enzymes involved in polyamine synthesis (*AtADC1-2* (**a**), *AtSAMDC1-4* (**b**), *AtSPDS1-2* (**c**), and *AtSPMS* (**d**)) during the direct formation of shoot meristem from lateral root primordia (at the organ conversion (48 h), the early (72 h) and late (96 h) shoot promeristem phases [7]). The mRNA levels of the UBIQUITIN 1 (At3G52590) and PP2A3 (At1G13320.1) genes were used for gene expression normalization. The mRNA level of the untreated root tissue was used as control (relative mRNA level: 1). Data were averaged from three independent biological experiments with three technical replicates each. Standard errors are shown on the columns. The significance of difference between sets of data was determined by one-way analysis of variance (ANOVA) following Duncan’s multiple range tests; a *p*-value of less than 0.05 was considered significant as indicated by different letters.

In Arabidopsis, the first step of polyamine biosynthesis, which results in putrescine from arginine or ornithine, is exclusively catalyzed by ARGININE DECARBOXYLASE. This enzyme exists as two isoforms (AtADC1 and AtADC2). *AtADC1* is constitutively expressed while *AtADC2* is responsive to stress stimuli [46,47,48]. Interestingly, during developmental processes both AtADC1 and AtADC2 are essential [17,49]. In our system, expression of *AtADC1* increased ≈12-fold after 48 h cytokinin induction (Figure 2a). This is the time when the LRPs gradually convert to shoot promeristems (“converting organ” state) [7]. During the appearance of early and late shoot promeristems, the mRNA level of AtADC1 remained high, but the expression of *AtADC2* gene was also enhanced at a lower degree (Figure 2a). Since the Put content was only slightly enhanced after 48 h cytokinin induction in contrast to Spd (Figure 1a), it is reasonable to assume that the Put produced by ADC1 (or ADC2) was quickly metabolized by DAO or converted to spermidine by SPERMIDINE SYNTHASE (SPDS).

Conversion of Put to Spd or Spm by the repetitive addition of the aminopropyl moieties from decarboxylated *S*-adenosylmethionine (dSAM) is mediated by SPDS and SPERMINE SYNTHASE (SPMS), respectively.

Formation of dSAM is regulated from SAM by the action of SAMDC. Among the five paralogs of *AtSAMDC* genes, *AtSAMDC4* gene was shown to have auxin-regulated promoter elements together with high expression in seedlings, not like the other *AtSAMDC* genes [50]. The expression of *AtSAMDC2* and *AtSAMDC4* was enhanced during the root-to-shoot meristem conversion and shoot meristem establishment process while the mRNA levels of *AtSAMDC1* and *AtSAMDC3* were reduced compared to the control (Figure 2b). The *AtSAMDC2* gene exhibited an especially high expression at the latest investigated state (96 h). Downregulation of the *AtSAMDC2* gene expression by RNA interference was reported to decrease the shoot regeneration response [44] indicating the importance of PA synthesis during the process. Increased SAMDC activity and Spd level correlated with higher embryogenic response in alfalfa [51].

In Arabidopsis, SPDSs are encoded by two genes, *AtSPDS1* and *AtSPDS2*, which are essential for several developmental processes, such as embryo development and plant survival [52]. After 48 h cytokinin induction, but mainly during the formation of shoot promeristem, the mRNA level of *AtSPDS1*, but mainly that of *AtSPDS2*, were elevated (Figure 2c). These results are in accordance with the 3-fold increase of Spd levels in the cytokinin-induced samples in comparison to the control (Figure 1a).

*SPMS* expression did not change during the converting organ state but slightly increased after 72 h of cytokinin induction (Figure 2d). In agreement, the Spm content did not increase during the shoot promeristem establishment process (Figure 1a).

Altogether, these data suggest that the augmented synthesis and conversion of Put to Spd correlates with direct shoot organogenesis from Arabidopsis roots. 

### 3.3. Ectopically Expressed AtPAO5 Enhances the Direct Conversion of LRPs to SMs

Besides the interference of PA synthesis with ethylene production [16], polyamines might influence morphogenesis via their products of degradation [35,45] or conversion [9]. The PA catabolic enzymes, POLYAMINE OXIDASEs (PAOs) catabolize Spd and Spm and produce 1,3-diaminopropane (DAP) and H_2_O_2_ [26]. PAO enzymes also function to convert tetraamine to triamine and/or triamine to diamine (backconversion reactions) [9,30].

**Figure 3:**
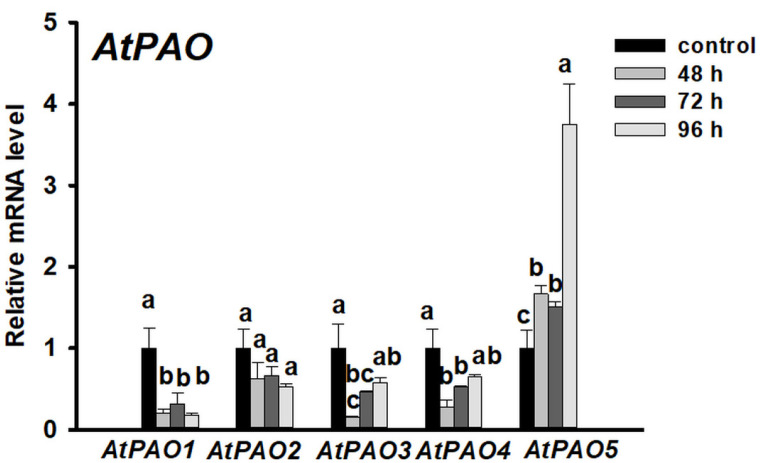
Expression of polyamine oxidase (PAO) genes during direct shoot organogenesis on Arabidopsis roots. Relative mRNA levels of *AtPAO1*, *AtPAO2*, *AtPAO3*, *AtPAO4*, and *AtPAO5* genes after 48, 72 and 96 h of cytokinin induction representing the organ conversion (48 h), the early (72 h) and late (96 h) shoot promeristem phases [7]. The mRNA levels of the UBIQUITIN 1 (At3G52590) and PP2A3 (At1G13320.1) genes were used for gene expression normalization. The mRNA level of the untreated root tissue was used as control (relative mRNA level: 1). Data were averaged from three independent biological experiments with three technical replicates each. Standard errors are shown on the columns. The significance of difference between sets of data was determined by one-way analysis of variance (ANOVA) following Duncan’s multiple range tests; a *p*-value of less than 0.05 was considered significant as indicated by different letters.

**Figure 4:**
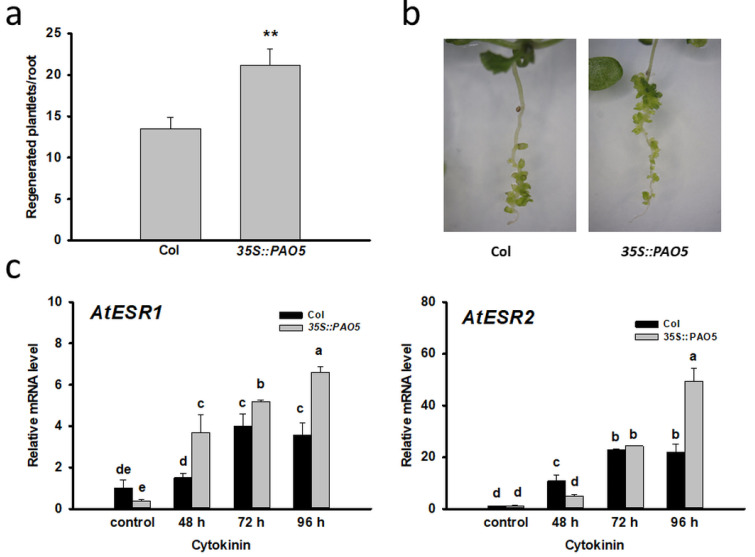
Ectopic overexpression of AtPAO5 enhances the conversion of lateral root primordia into shoots. (**a**) Shoot regeneration of seedling roots of WT (Col) and *AtPAO5* overexpressing transgenic (*35S::PAO5*) plants 10 days after cytokinin induction. Data are means ± SE of three biological replicates with 20 seedlings each. For significance analysis Student’s *t*-test was used (** *p* ≤ 0.01). (**b**) Representative images of regenerating seedlings. (**c**) Relative mRNA levels of *AtESR1* and *AtESR2* genes in wild type (Col) and *AtPAO5*-overexpressing (*35S::PAO5*) seedling roots after 48, 72 and 96 h of cytokinin induction representing the organ conversion (48 h), the early (72 h) and late (96 h) shoot promeristem phases [7]. The mRNA levels of the UBIQUITIN 1 (At3G52590) and PP2A3 (At1G13320.1) genes were used for gene expression normalization. The mRNA level of the untreated root tissue was used as control (relative mRNA level: 1). Data were averaged from three independent biological experiments with three technical replicates each. Standard errors are shown on the columns. The significance of differences between sets of data was determined by one-way analysis of variance (ANOVA) following Duncan’s multiple range tests; a *p*-value of less than 0.05 was considered significant as indicated by different letters.

In Arabidopsis five AtPAOs (AtPAO1, AtPAO2, AtPAO3, AtPAO4, AtPAO5) are involved in polyamine catabolism among which only the expression of *AtPAO5* increased after 48 h of cytokinin induction and further augmented during the formation of early and late shoot promeristem (Figure 3). To verify the involvement of AtPAO5 in direct shoot organogenesis, *AtPAO5*-overexpressing transgenic plants (*35S:PAO5*) were investigated for shoot regeneration efficiency. We found that *35S:PAO5*-expressing seedlings had more regenerates/root (Figure 4a,b) and expressed the *ENHANCER OF SHOOT REGENERATION 1* and *2* (*ESR1* and *2*) genes at higher level than the WT ones (Figure 4c). The expression of the genes coding for the ESR1,2 transcription factors was shown to be strongly associated with in vitro shoot organogenesis [53,54] and thus can serve as marker for the conversion process. These data indicate that *AtPAO5* expression promotes the direct conversion of LRPs to SMs. AtPAO5 is considered to be unique in comparison to the other Arabidopsis PAOs having a dehydrogenase rather than an oxidase activity [9,30]. Therefore, in order to test whether the effect of *35S:PAO5* expression on direct shoot regeneration is specific, transgenic seedlings overexpressing the *35S:PAO2* gene [55] were also tested for the shoot regeneration rate of cytokinin-treated roots. No difference in the LRP-to-SM conversion frequency of WT and 35S:PAO2 seedlings was detected (Supplementary Figure S1).

**Figure 5:**
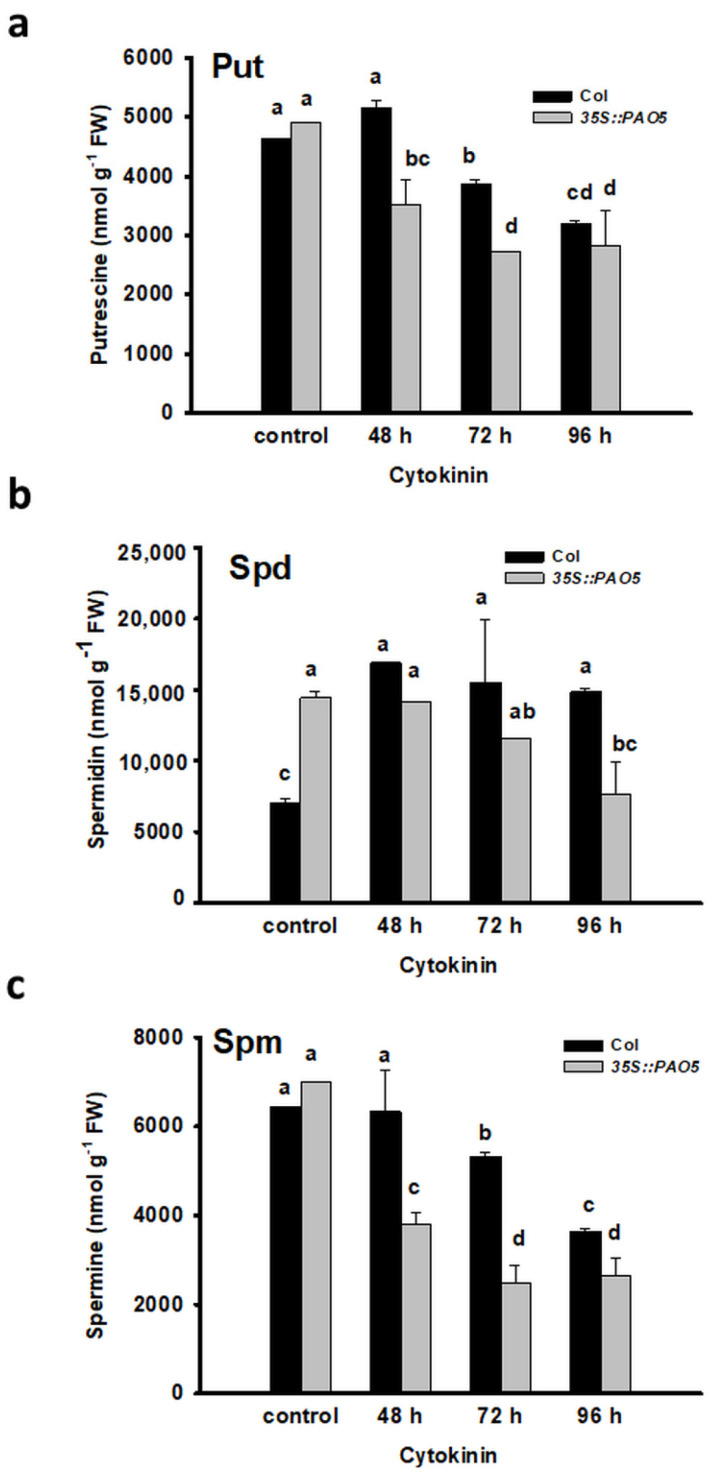
AtPAO5 overexpression influences the PA homeostasis in seedling roots before and during the conversion of lateral root primordia into shoots. Putrescine (Put) (**a**), spermidine (Spd) (**b**) and spermine (Spm) (**c**) contents were determined in 100 mg of root samples of wild type (Col) and transgenic (35S:PAO5) seedlings after 48, 72 and 96 h cytokinin treatment repre-senting the organ conversion (48 h), the early (72 h) and late (96 h) shoot promeristem phases [7]. Data are means ± SE of three biological replicates with 20 technical replicates each. Different let-ters indicate significant differences of Duncan’s multiple comparisons (*p* < 0.05).

Measuring the polyamine contents in the 35S:PAO5 roots before and during the direct shoot regeneration process revealed that the initial level of Spd, but not that of Put or Spm, was significantly higher in the transgenic roots than in the control (Figure 5). Following the cytokinin-treatment, the level of all three plyamines gradually decreased at a higher rate in the 35S:PAO5 than in the WT roots (Figure 5) indicating that *AtPAO5* overexpression influenced the polyamine homeostasis of seedling roots.

**Figure 6:**
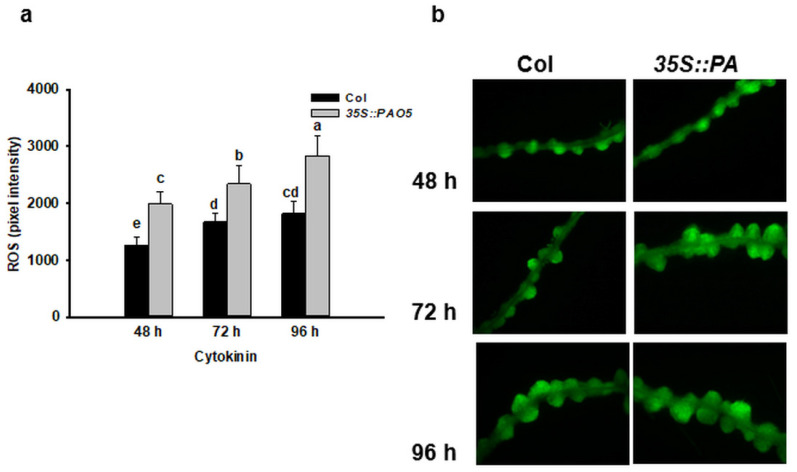
ROS level influenced by 35S::PAO5 expression correlates with the process of direct shoot meri-stem formation. Representative diagram (**a**) and images (**b**) of 2,7-dichlorodihydro-fluorescein diacetate (H2DC-FDA)-derived DCF fluorescence in WT (Col) and transgenic (35S::PAO5) seed-ling roots after 48, 72 and 96 h cytokinin treatment representing the organ conversion (48 h), the early (72 h) and late (96 h) shoot promeristem phases [7]. Scale bars = 100 µm. Data are means ± SE of three biological replicates with twenty technical replicates each. Different letters indicate significant differences of Duncan’s multiple comparisons (*p* < 0.05).

In addition to the altered polyamine content, the ROS level was found to be different in the LRPs of transgenic 35S::PAO5 seedlings compared to the WT ones (Figure 6). Lower ROS accumulation in the *atpao5* seedlings has been reported [56] supporting the view that AtPAO5 modify the ROS homeostasis. H_2_O_2_ as the product of PA catabolism was found to be essential for the indirect formation of somatic embryos in cotton [27] as well as for lateral root formation in soybean [57]. H_2_O_2_ is linked to various plant morphogenic processes [58,59,60], including de novo shoot regeneration [61,62]. H_2_O_2_ exerts its effect on shoot regeneration in a context and concentration dependent way: although it inhibits shoot formation at higher doses, it promotes the initial phase of de novo organogenesis at low concentrations [44,55]. It was hypothesized that PAs may influence plant regeneration mediated by H_2_O_2_ formed because of their oxidation e.g., by polyamine oxidases [34,44]. Why the overexpresion of *AtPAO5* but not that of *AtPAO2*, the activity of which is also associated with ROS production, enhanced direct shoot regeneration is not known.

It has to be mentioned, however, that the two enzymes have different cellular localization (AtPAO5 cytoplasmic [9], AtPAO2 peroxisomal [30]) and AtPAO5 is unique among the five Arabidopsis PAOs considering its high affinity for t-Spm as a substrate [9]. AtPAO5, was shown to be involved in the maintenance of t-Spm homeostasis that is required for normal plant development: the *atpao5* mutant lacking t-Spm oxidation and the *acl5* mutant lacking t-Spm synthesis both exhibit growth defects [8,9]. Therefore, maintaining t-Spm homeostasis was attributed as the primary function of AtPAO5 [9]. Nevertheless, AtPAO5 was also reported to serve as a spermine oxidase/dehydrogenase in *35S:PAO5*-expressing transgenic plants [31]. It was hypothesized that although the preferential in vivo substrate of AtPAO5 is t-Spm, if ectopically overexpressed it can efficiently access and metabolize Spm [9].

Transgenic Arabidopsis plants with up- or downregulated ARABIDOPSIS FLAVIN-CONTAINING AMINE OXIDASE 1 (AtFAO1) (AtFAO1 is an earlier designation of AtPAO5) expression were shown to exhibit strongly altered indirect shoot regeneration abilities from root explants [45]. While the regeneration efficiency of root explants with downregulated AtFAO1/AtPAO5 activity was elevated, those ones with upregulated activity were only poorly regenerative [45]. This latter observation seems to be contradictory to our results, since we found increased conversion of LRPs to shoots in the line overexpressing *AtPAO5*. This contradiction might be due to the excision of the seedling roots followed by auxin-induced callus formation during the indirect process studied by Lim et al. (2006) [45], while we used intact seedlings treated by high concentration of cytokinin in the direct shoot regeneration pathway. AtPAO5 was shown to influence the interplay of auxin and cytokinin during xylem differentiation [8]. Although shoot meristem formation may follow the same steps during the direct and indirect pathways, the difference in the hormonal and redox environment of surrounding tissues (callus or LRP) might result in altered sensitivity of the initial events towards the products/consequences of ectopic AtPAO5 activity.

## 4. Conclusions

Polyamines have already been shown to control plant morphogenesis and in vitro plant regeneration in several experimental systems. Here we provide evidence that they are also involved in the cytokinin-induced direct (without intervening callus formation) conversion of lateral root primordia (LRPs) of intact seedlings into shoot meristems. This process that includes the correct temporal and spatial organization of cell divisions and cell fate decisions was found to correlate with increased accumulation of spermidine (Spd). In agreement, the expression of genes related to Spd synthesis (*AtADC1,2*, *AtSAMDC2,4*, *AtSPDS1,2*) and backconversion of t-Spm/Spm to Spd (*AtPAO5* but not *AtPAO1-4*) was increased following the cytokinin treatment. In agreement, the ectopic overexpression of *AtPAO5* in transgenic seedlings increased the Spd level and the shoot regeneration rate of roots. Furthermore, exogenous Spd treatment could also promote the conversion of lateral root primordia to shoot meristems. Interestingly, this observation is in contradiction with reports about indirect shoot organogenesis from excised Arabidopsis root segments where the overexpression of *AtPAO5* reduced the spermidine level and the regeneration potential. This indicates that PAs and PA metabolism affect the shoot regeneration process in Arabidopsis in a context-dependent manner.

Ectopic expression of *AtPAO5* also resulted in elevated ROS level in the LRPs undergoing conversion to shoot meristems and the contribution of this ROS to regeneration enhancement cannot be excluded. It is to be noted that although AtPAO1-4 are also capable of Spm backconversion and associated ROS production, only *AtPAO5* was found to show elevated expression in the investigated shoot regeneration pathway, and the ectopic expression of *AtPAO5* but not that of *AtPAO2* could increase the regeneration efficiency. These observations implicate a specific role for AtPAO5 in the process. Since AtPAO5 was shown to have specifically high affinity for t-Spm, one can suppose that it may also influence shoot regeneration controlling t-Spm homeostasis. This possibility, however, still needs to be experimentally addressed.

## Data Availability

Data sharing is not applicable to this article.

## Supplementary Materials

The following are available online at https://www.mdpi.com/2223-7747/10/2/305/s1, Supplementary Figure S1 AtPAO2 overexpression didn’t influence the conversion rate of lateral root primordia into shoots. Supplementary Table S1. Sequences of the oligonucleotide primers used in the qPCR experiments.
