# Supplementary material for: Polyamine Metabolism Is Involved in the Direct Regeneration of Shoots from Arabidopsis Lateral Root Primordia
Source: Plants (Basel). 2021 Feb 5;10(2):305. doi: 10.3390/plants10020305 (PMC7915173; doi:10.3390/plants10020305)
Supplement: Supplementary file 1 [file plants-10-00305-s001.zip › New folder/Suppl Fig1.pptx]

## Slide 1
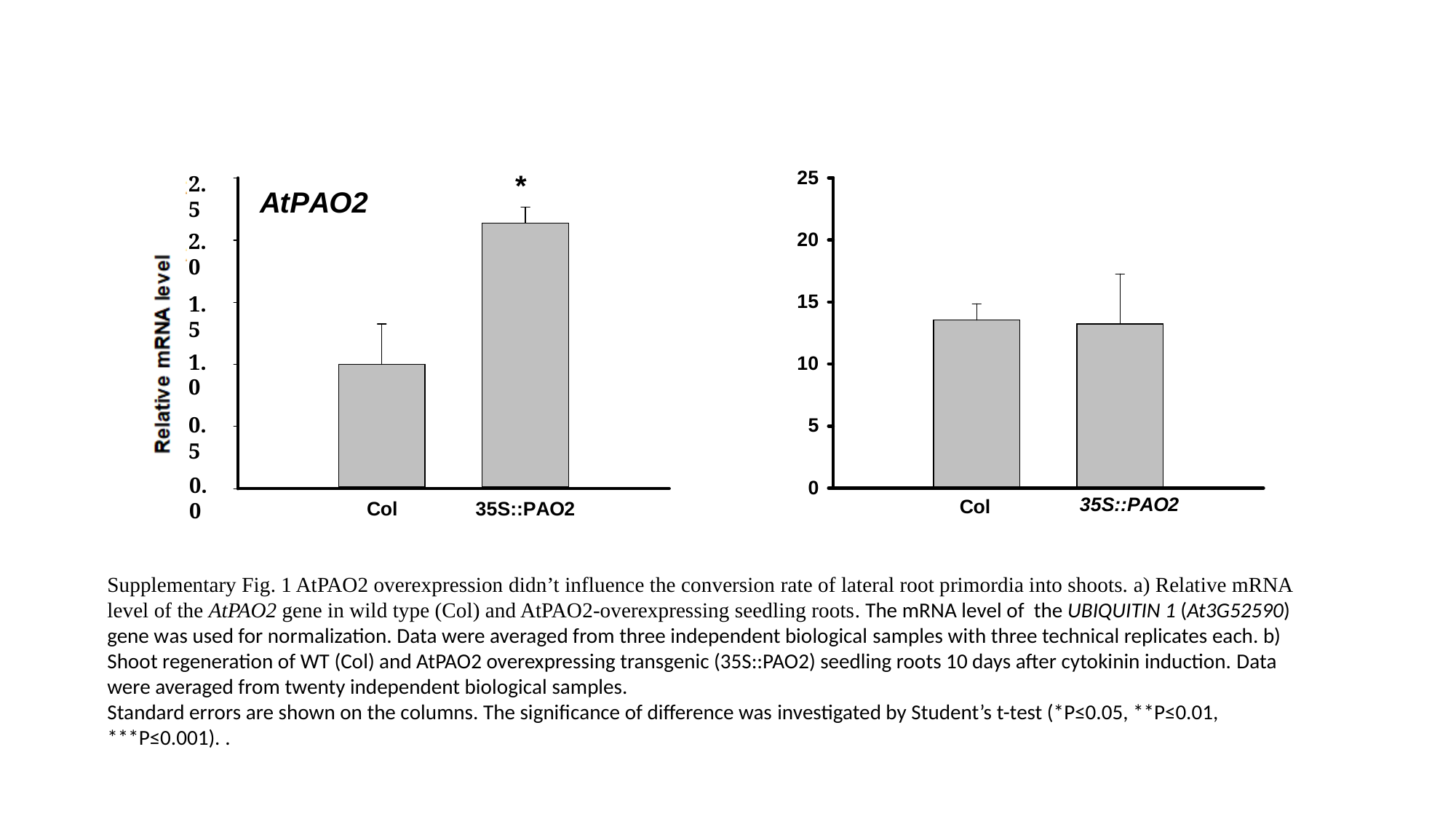

2.5
2.0
1.5
1.0
0.5
0.0
Supplementary Fig. 1 AtPAO2 overexpression didn’t influence the conversion rate of lateral root primordia into shoots. a) Relative mRNA level of the AtPAO2 gene in wild type (Col) and AtPAO2-overexpressing seedling roots. The mRNA level of the UBIQUITIN 1 (At3G52590) gene was used for normalization. Data were averaged from three independent biological samples with three technical replicates each. b) Shoot regeneration of WT (Col) and AtPAO2 overexpressing transgenic (35S::PAO2) seedling roots 10 days after cytokinin induction. Data were averaged from twenty independent biological samples.
Standard errors are shown on the columns. The significance of difference was investigated by Student’s t-test (*P≤0.05, **P≤0.01, ***P≤0.001). .
