# Supplementary material for: Polyamine Metabolism Is Involved in the Direct Regeneration of Shoots from Arabidopsis Lateral Root Primordia
Source: Plants (Basel). 2021 Feb 5;10(2):305. doi: 10.3390/plants10020305 (PMC7915173; doi:10.3390/plants10020305)
Supplement: Supplementary file 1 [file plants-10-00305-s001.zip › New folder/Suppl Table1.docx]

Supplementary Table 1. Sequences of the oligonucleotide primers used in the qPCR experiments

| F: 5' - GAGTCCACAATCTGTTCGGTG - 3'  R: 5' - GTTGTTGTTGTTCCTCGGCT - 3' | *At ADC1 (AT2G16500)* |
| --- | --- |
| F: 5' - GTGGTGGTTTGGGGATTGAC- 3'  R: 5' - CGGGAGTTGCTTGATGAACC- 3' | *At ADC2 (AT4G34710)* |
| F: 5' - TCCGCTTCACTTCACACCAA - 3'  R: 5' - GCCGCCGTTATCATCCTCTT- 3' | *At SPDS1 (AT1G23820)* |
| F: 5' - CTGATCCAATCGGTCCAGCA - 3'  R: 5' - GGAACACTGGTCCAAGCGTA- 3' | *At SPDS2 (AT1G70310)* |
| F: 5' - TTTTCGGAACCTCACCCTCG - 3'  R: 5' - CTTTCCGTACGTGGCTGACT - 3' | *At SPMS (AT5G53120*) |
| F: 5' - CTACCGCCTCCGTCATCATC - 3'  R: 5' - ACTCTTTACCACCGACACCG- 3' | *At PAO1 (AT5G13700)* |
| F: 5' - AGAGGCTTAGGGTTCCAGTG - 3'  R: 5' - ATCCAACTCACCATACCGCT- 3' | *At PAO2 (AT2G43020)* |
| F: 5' - GGATGAGCAGGACGAAGACA- 3'  R: 5' - GATCCCAACACTTTGCCGAG - 3' | *At PAO3 (AT3G59050)* |
| F: 5' - TTCCGCCGCAGTTGGTTA- 3'  R: 5' - CGCCATCCCTTCTTGCCT- 3' | *At PAO4 (AT1G65840)* |
| F: 5' -TCGGTGGAAGCCCTGTTTAT - 3'  R: 5' -CGGCGTCGGATTGAGAGAT - 3' | *At PAO5 (AT4G29720)* |
| F: 5’ - CAACACCCTTGACCTTAGCC - 3’  R: 5’ - GCTTGTATGAGTTCGCTCCA - 3’ | *AtSAMDC1 (AT3G02470.1)* |
| F: 5’ TACTTGATGGGCAATGACGA- 3’  R: 5’ -TGTTGGCAGAAGAAGCAGAG - 3’ | *AtSAMDC2 (AT5G15950.1)* |
| F: 5’ - CGAGGTATTGGCGTCTGATT- 3’  R: 5’ - GGTGAGCAACATTCAACAGTCT- 3’ | *AtSAMDC3 (AT3G25570.1)* |
| F: 5’ - CTCTACGACAACGGTCACGA - 3’  R: 5’ - CACTTGGACGGAAAACATCA - 3’ | *AtSAMDC4 (AT5G18930.1)* |
| F: 5’ - TCCTCCTTCTCCACCAACAC - 3’  R: 5’ - AACGATAAACCACCGTCTGC - 3’ | *AtESR1*  *(AT1G12980.1)* |
| F: 5’ - GCTGACTTCCATGTCGAAGGA- 3’  R: 5’ - TCTGCTGCATCTTAGCTGAATC - 3’ | *AtESR2*  *(AT1G24590)* |
| F: 5’ - CGCAAGTGCTATGCTCGTCTT - 3’  R: 5’ - GCCTCAACTGGTTGCTGTGA - 3’ | *UBIQUITIN1* (*AT3G52590*) |
| F: 5’ - ATTCCGATAGTCGACCAAGC -3’  R: 5’ - AACATCAACATCTGGGTCTTCA - 3’ | *PP2AA3*  *(AT1G13320.1)* |
